# Telemedicine: Can In-Person Pre-treatment Communication be Expanded by Video Consultation?

**DOI:** 10.1007/s00270-019-02337-z

**Published:** 2019-09-06

**Authors:** S. Guhl, L. Linngrön, B. Rosenberg, N. Hosten, M. Kirsch

**Affiliations:** Department of Diagnostic Radiology and Neuroradiology, Universitätsmedizin Greifswald, Ferdinand-Sauerbruch-Straße, 17475 Greifswald, Germany

To the Editor,

Informed consent for radiological or other interventions should give a patient sufficient time to make an informed decision. Currently, patients typically have to be present, in-person, to be briefed about procedures and an extra appointment is often necessary. While in an urban setting this is mostly just a nuisance, in a rural area, similar to ours, it may not be possible at all for patients with limited access to transport. In some countries, teleconsultation via videoconference has proved beneficial in comparable situations [1].

**Fig. 1:**
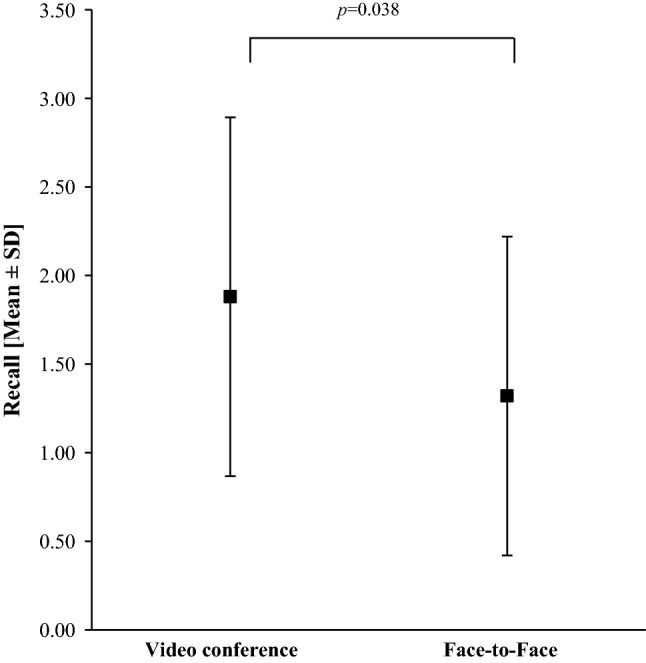
Recall was higher for videoconference briefings than for face-to-face briefings

**Fig. 2:**
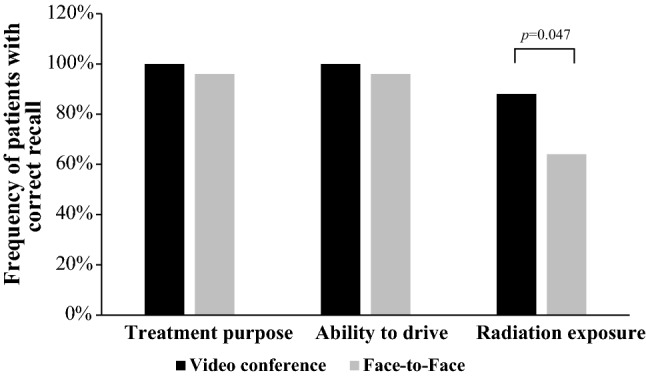
Recall of items explained in briefings

We performed a pilot study, approved by the ethical committee of our university. Fifty patients were 1:1 randomized and one group was briefed face-to-face, the other via videoconferencing. With our hospital being located in a rural area, patients travel 50.2 km to our department for periradicular therapy (mean: range 1–110 km). Thirty-two percent of study patients already used videoconferencing Apps such as Skype^®^ or Facetime^®^ in their private lives. Patients provided written consent firstly to the intervention itself and secondly to the pilot study presented here. They were informed that participation in the study was voluntary. Patients were referred for CT-guided periradicular or facet join infiltration aimed at reducing chronic back pain. Groups did not show significant differences regarding the age (*t*(48) = − 1.827, *p *= 0.074, *n *= 50) or distribution between the sexes (*χ*^***2***^(1) = 0.89, *p *= 0.765, *n *= 50) (57 years vs. 64 years and 68% males vs. 61% males for videoconference vs. in-person, respectively). Interventions were performed by a radiologist supported by technical assistants. Both groups received the same pre-treatment discussion by the performing radiologist [2], followed by the legally required face-to-face briefing for the videoconferencing group later. To compare the effectiveness of both options, a questionnaire was read to patients in a telephone call by a blinded study nurse 24 h after the briefing [3]. A total of eight questions covered atmosphere and necessity of information, quality of the doctor/patient relationship and the feeling of being taken seriously by the physician. The remaining questions covered the items summarized in Figs. 1 and 2.

Patients who received the pre-treatment briefing by videoconference remembered significantly more (Mann–Whitney *U* test: *U *= 210.000, *p *= 0.038, *r *= 0.2932) of the mentioned side effects compared to patients who received the pre-treatment briefing in-person (Fig. 1). Further, the recall of radiation exposure was significantly higher when communicated in a videoconference (*χ*^***2***^(1) = 3.947, *p *= 0.047, *n *= 50, *φ *= − 0.281, Fig. 2). For patient satisfaction with pre-treatment communication and the other variables related to knowledge acquisition, no significant differences emerged.

Preoperative discussion by videoconferencing was equal to, or better than, face-to-face discussion. We assume that patients easily focus on a monitor, and distraction is thus reduced [4]. While there may be extra costs to cover the equipment, the process of informing patients about procedure may actually be facilitated (less logistical effort, patients may be given a specific time window for the call). Briefings via videoconference could be saved, with additional viewings made available to patients. Additional information material may also be provided easily.

In our opinion, it is worthwhile to evaluate “informed consent to treatment in low population density areas by teleconsultation” in larger studies. A higher number of participants are necessary as effect sizes were small; more realistic scenarios with clinic to home videoconferencing should be employed; improving the process of blinding of study nurses, if possible, as patients tended to mention the videoconference in interviews; legal aspects (saving the interviews digitally; use of electronic devices for getting informed consent in one study group) must be clarified beforehand, and endpoints of studies should be chosen in a way that validated questionnaires can be used.
